# Relationship Between IL6/IL10 Serum Concentrations and Organ Function in Critically Ill Patients Based on Sepsis: A Prospective Study

**DOI:** 10.1002/iid3.70414

**Published:** 2026-04-09

**Authors:** Guang‐Jian Wang, Hui Lian, Hong‐Min Zhang, Ye‐Cheng Liu, Xiao‐Ting Wang

**Affiliations:** ^1^ Department of Health Care, Peking Union Medical College Hospital Chinese Academy of Medical Sciences & Peking Union Medical College Beijing China; ^2^ Emergency Department, Peking Union Medical College Hospital Chinese Academy of Medical Sciences & Peking Union Medical College Beijing China; ^3^ Department of Critical Care Medicine, Peking Union Medical College Hospital Chinese Academy of Medical Sciences & Peking Union Medical College Beijing China

**Keywords:** IL6/IL10, inflammatory response, organ dysfunction, sepsis

## Abstract

**Background:**

Critical illnesses, particularly sepsis with accompanying organ dysfunction, significantly increase patient mortality. The imbalance between pro‐inflammatory and anti‐inflammatory responses is closely linked to the onset and progression of organ dysfunction in critical conditions. The ratio of interleukin‐6 (IL6) to interleukin‐10 (IL10) is a useful indicator of the pro‐ and anti‐inflammatory status.

**Purpose:**

To investigate the correlation between IL6/IL10 and various outcomes, including organ function, in critically ill patients.

**Methods:**

This prospective study was conducted from 1 January to 31 May 2023 at Peking Union Medical College Hospital. Baseline characteristics, intensive care unit (ICU) parameters, laboratory test results, and outcome data were extracted from the electronic medical records system. Correlations between IL6/IL10 and various outcomes, including organ function, were analyzed.

**Result:**

In total, 208 patients were included in the study. Higher IL6/IL10 levels were associated with shorter in‐hospital stay, ICU stay, and mechanical ventilation time in both univariate and multivariate models. The β values and 95% confidence intervals in the multivariate model were −0.526 (− 0.684, −0.369), −0.183 (− 0.245, −0.121), and −1.510 (− 2.565, −0.456), respectively. Significant positive associations between IL6/IL10 and all organ functions except the platelet count and PaO_2_/FiO_2_ ratio were found. Sensitivity analyses in the sepsis group revealed significant correlations with nearly every organ function (except liver function) in patients without tumors and those who underwent surgery.

**Conclusion:**

Our study showed that in critically ill patients, particularly those with sepsis, IL6/IL10 is significantly associated with outcomes such as organ dysfunction (including coagulation, renal, cardiac, and pulmonary function). These findings are hypothesis‐generating, suggesting that IL6/IL10 may serve as a valuable indicator associated with inflammation and severity changes in critical illness.

## Graphical abstract

Our study found that in critically ill patients, especially septic patients, IL6/IL10 is significantly associated with outcomes including organ dysfunction (including coagulation, renal, heart, and lung), which can be considered a good indicator for assessing inflammation status and predicting severity changes in critical illness. Abbreviation: IL, interleukin. The picture was drawn by Figdraw.

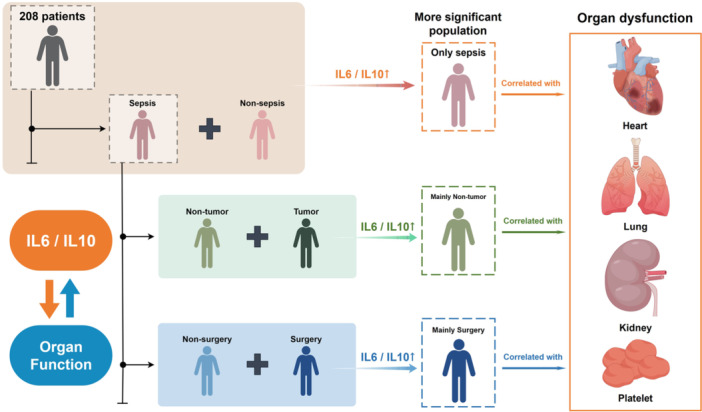

## 1 Introduction

Critical illnesses may share common molecular mechanisms of damage. Infections trigger the recognition of pathogen‐associated molecular patterns, while other conditions, such as trauma, produce damage‐associated molecular patterns. Both mechanisms lead to the release of pro‐inflammatory and anti‐inflammatory factors, which can drive the host response away from homeostasis in two opposing directions—excessive inflammation and immunosuppression. However, current immunological understanding characterizes sepsis not merely as a sequential progression from pro‐ to anti‐inflammatory phases, but rather as a highly complex, non‐linear, and compartmentalized process. Concurrent hyperinflammation and profound immunosuppression often coexist, driven by distinct immune endotypes and phenotypes. This imbalance between pro‐inflammatory and anti‐inflammatory responses can contribute to the onset and progression of critical illnesses [1, 2]. Sepsis, a classic example of a critical illness, is a leading cause of death among patients in intensive care units (ICUs), making it a significant public health issue worldwide [3]. According to the Sepsis‐3.0 definition, sepsis is a life‐threatening organ dysfunction caused by a dysregulated host response to infection [4, 5]. Among them, the inflammatory response plays a key role in the host response [4].

Sepsis and other critical illnesses frequently lead to organ dysfunction, which is closely linked to both disease severity and prognosis [6, 7]. Studies have shown that patients with organ dysfunction experience longer ICU stays, higher rates of ICU‐acquired weakness, and increased mortality relative to patients without organ dysfunction [8, 9]. The key clinical challenge is determining whether critically ill patients currently have or will develop organ dysfunction. As researchers continue to investigate the pathogenesis of organ dysfunction, the inflammatory response has emerged as a critical factor [10, 11]. The excessive release of various inflammatory factors, often referred to as a “cytokine storm” [12], leads to a systemic imbalance between pro‐inflammatory and anti‐inflammatory responses and thus increases the likelihood of organ dysfunction in critically ill patients [13].

Interleukin‐6 (IL6) and interleukin‐10 (IL10) are classical pro‐inflammatory and anti‐inflammatory markers, respectively. The blood concentrations of these cytokines reflect the intensity of the inflammatory response. The IL6/IL10 ratio is used to assess the balance between pro‐inflammatory and anti‐inflammatory responses: an increased ratio suggests a stronger pro‐inflammatory response, while a decreased ratio indicates a stronger anti‐inflammatory response [14]. Although the IL6/IL10 ratio has been linked to organ dysfunction in trauma patients, evidence supporting similar associations in patients with sepsis is limited [15, 16]. Therefore, further investigation into the relationship between the IL6/IL10 ratio and organ function parameters in septic patients is warranted.

In this study, we hypothesized that the serum IL6/IL10 ratio in critically ill patients, particularly those with sepsis, may be indicative of organ function. We first analyzed the correlation between the IL6/IL10 ratio and various organ function parameters. Next, we assessed the association of the IL6/IL10 ratio with clinical outcomes. Finally, we performed sensitivity analyses to explore the correlation between the IL6/IL10 ratio and organ function in different subgroups of patients with sepsis.

## 2 Methods and Materials

### 2.1 Study Design and Research Population

**Figure 1:**
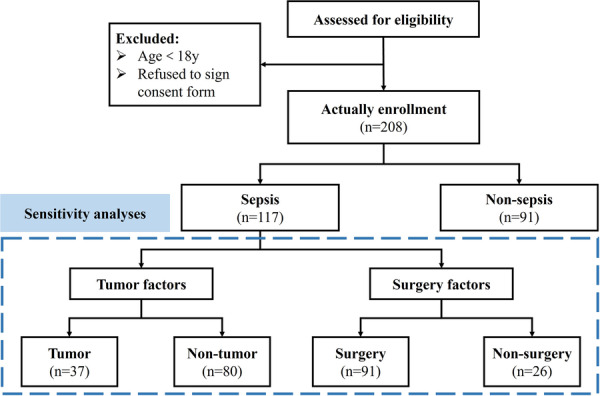
Flowchart of the study.

This prospective study was conducted from 1 January to 31 May 2023 at Peking Union Medical College Hospital, a leading tertiary hospital in China. We included patients aged ≥ 18 years who were admitted to the ICU. The study was approved by the local ethics committee (Ethical Approval Number: I‐22PJ1072), and written informed consent was obtained directly from the patients or their relatives. Patients aged < 18 years and those who refused to provide written informed consent were excluded. The entire research process is illustrated in Figure 1. Participants were categorized into non‐sepsis and sepsis groups based on the third international consensus definitions for Sepsis‐3.0 [5]. All clinical data and analytical methods are available from the corresponding author upon reasonable request.

### 2.2 Data Collection

We collected baseline characteristics, ICU parameters, laboratory test results, and outcome data from the electronic medical records system. Baseline characteristics included the medical history (such as tumor status) and surgical history. ICU parameters primarily consisted of heart rate, mean arterial pressure, Acute Physiology and Chronic Health Evaluation (APACHE) II score, norepinephrine dosage, and lactate levels, all recorded at the time of admission. Inflammatory biomarkers, including IL6, IL10, higher hypersensitive C‐reactive protein, and procalcitonin were also collected at admission. The IL6 and IL10 levels were measured using an immunofluorescence‐based flow cytometry assay (FACScalibur, BD Biosciences, San Jose, CA, USA) with specific reagent kits (Saiji Biotech, Nanchang, China).

The worst organ function during the patient's ICU stays (from admission to discharge), and the outcomes were recorded. Organ function measures included liver function (alanine aminotransferase [ALT]), renal function (creatinine and blood urea nitrogen [BUN]), coagulation function (platelet count [PLT]), cardiac function (cardiac troponin I [cTnI] and N‐terminal pro‐B‐type natriuretic peptide [NT‐proBNP]), and respiratory function (PaO_2_/FiO_2_ ratio).

Data extraction was performed independently by Wang G and Lian H, who cross‐checked the data using a standardized collection process. The data were then reviewed and confirmed by Liu Y and Wang X.

### 2.3 Endpoints

The primary endpoints were clinical outcomes, including mechanical ventilation time (MVt), ICU length of stay, and overall length of hospital stay. The secondary endpoints included various measures of organ function.

### 2.4 Statistical Analysis

Because none of the continuous variables followed a normal distribution based on the Kolmogorov–Smirnov test for normality, they are presented as median (25th percentile, 75th percentile). Categorical variables are presented as number (percentage). The *χ* ^2^ test was used to compare categorical variables, while continuous variables with a non‐normal distribution were analyzed using the Mann–Whitney *U* test. A generalized linear model was applied for both univariate and multivariate analyses. To ensure model robustness, variance inflation factors were calculated to assess potential multicollinearity between IL6, IL10, and other inflammatory markers prior to the generalized linear model analysis. To amplify the estimated effect, IL6/IL10 values were divided by 100 before being included in the regression model. Age, sex, and APACHE II score were adjusted for in the multivariate model. A two‐tailed *p*‐value of < 0.05 was considered statistically significant in all analyses. All statistical analyses were performed using R software, version 4.2.0 (http://www.R-project.org/). Figure 1 was created using WPS (Kingsoft, Beijing, China), while all other figures were generated with GraphPad Prism, version 10.0 (GraphPad Software, San Diego, CA, USA).

## 3 Results

In total, 208 patients were included after strict screening based on the inclusion and exclusion criteria. Among them, 117 patients were diagnosed with sepsis, while 91 were categorized into the non‐sepsis group. Most of the patients with sepsis (59.0%) were male. Nearly half of the study population (*n* = 98) had a tumor diagnosis, and the majority (85.1%) of the 208 patients underwent surgical procedures (Figure 1). Only 44 of the patients were diagnosed with diabetes before and few of them were with autoimmune disease. The median age of the study population was 60 years. Most non‐surgical patients were diagnosed with sepsis during their ICU stay. In the sepsis group, the heart rate, lactate level, norepinephrine dosage, and APACHE II scores were all higher than those in the non‐sepsis group.

In terms of outcomes, the mortality was significantly higher in the sepsis group; none of the patients in the non‐sepsis groups died. Although the overall length of hospital stay was not significantly different between the two groups, the length of ICU stays and MVt were longer in the sepsis group (Table 1).

**Table 1:** Clinical characteristics of the study population.

	Total *N* = 208	Non‐sepsis *N* = 91(43.7%)	Sepsis *N* = 117(56.3%)	*p*‐value
	N(%)/Medium(25th, 75th)	N(%)/Medium(25th, 75th)	N(%)/Medium(25th, 75th)	
Baseline characteristics				
Age (years)	60 (50, 69)	59 (45, 69)	60 (53, 69)	0.106
Gender (%)				**< 0.001**
Female	110 (52.9%)	62 (68.1%)	48 (41.0%)	
Male	98 (47.1%)	29 (31.9%)	69 (59.0%)	
Surgery (%)				**< 0.001**
No	31 (14.9%)	5 (5.5%)	26 (22.2%)	
Yes	177 (85.1%)	86 (94.5%)	91 (77.8%)	
Diabetes (%)				0.669
No	164 (78.8%)	73 (80.2%)	91 (77.8%)	
Yes	44 (21.2%)	18 (19.8%)	26 (22.2%)	
Autoimmune Disease (%)				0.188
No	193 (92.8%)	82 (90.1%)	111 (94.9%)	
Yes	15 (7.2%)	9 (9.9%)	6 (5.1%)	
Tumor (%)				**< 0.001**
No	110 (52.9%)	30 (33.0%)	80 (68.4%)	
Yes	98(47.1%)	61 (67%)	37 (31.6%)	
Inflammation biomarkers				
IL6 (pg/mL)	157.9 (73.3, 460.5)	135.8 (61.2, 293.2)	208.8 (83.3, 529.6)	**0.019**
IL10 (pg/mL)	11.5 (6.1, 23.6)	9.8 (5.0, 17.8)	14.9 (7.3, 29.0)	**0.006**
hsCRP (mg/L)	81.0 (42.6, 165.7)	59.5 (22.8, 88.8)	94.3 (45.3, 191.8)	0.068
PCT (ng/mL)	0.4 (0.1, 1.8)	0.2 (0.1, 0.5)	0.9 (0.2, 5.0)	**0.002**
ICU Parameters				
HR (bpm)	91 (78, 104)	83 (74, 97)	94 (80, 109)	**0.002**
MAP (mmHg)	97 (86, 106)	98 (91, 105)	94 (84, 107)	0.087
Lac (mmol/L)	1.7 (1.0, 3.5)	1.5 (0.9, 2.2)	2.0 (1.1, 4.0)	**0.004**
NE (ug/kg/min)	0.1 (0.0, 0.3)	0.0 (0.0, 0.1)	0.1 (0.0, 0.4)	**< 0.001**
APACHE II (score)	14 (10, 17)	11 (9, 15)	16 (12, 19)	**< 0.001**
Outcomes				
Mortality (%)				0.004
No	198 (95.2%)	91 (100%)	107 (91.5%)	
Yes	10 (4.8%)	0 (0%)	10 (8.5%)	
LOS (days)	15 (10, 22)	14 (10, 20)	15 (11, 24)	0.064
ICU stay (days)	3 (2, 5)	2 (2, 3)	5 (3, 8)	**< 0.001**
MVt (hours)	13.5 (3, 46.5)	3 (1.5, 12)	37 (10, 83)	**< 0.001**
MV (%)				**< 0.001**
No	28 (13.5%)	20 (22.0%)	8 (6.8%)	
Yes	180 (86.5%)	71 (78.0%)	109 (92.2%)	

*Note:* Bold values indicate statistically significant *p* < 0.05.

Abbreviations: APACHE II, Acute Physiology and Chronic Health Evaluation II; ICU, intensive care unit; hsCRP, higher hypersensitive C‐reactive protein; HR, heart rate; IL, interleukin; LOS, length of stay; MAP, mean arterial pressure; MVt, mechanical ventilation time; NE, norepinephrine; PCT, procalcitonin.

**Figure 2:**
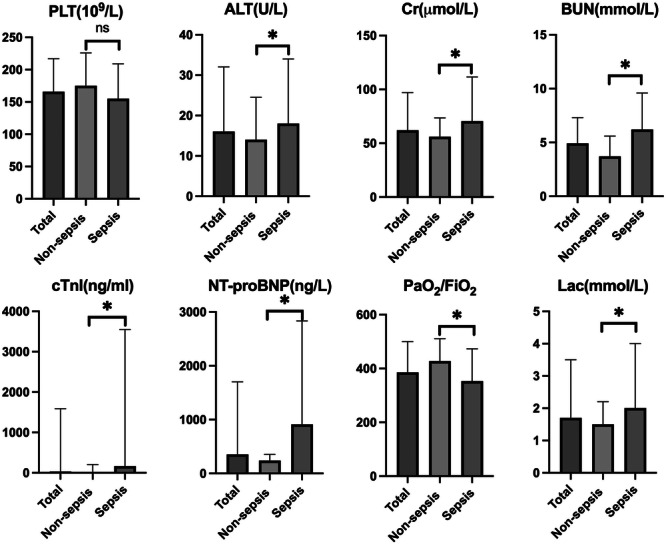
Organ function of the study population. ALT, alanine aminotransferase; BUN, blood urea nitrogen; Cr, creatinine; cTnI, cardiac troponin I; Lac, lactate; NT‐proBNP, N‐terminal pro‐B‐type natriuretic peptide; PLT, platelet count. The median values and 75th percentiles are shown. Outliers were removed. *p < 0.05.

Figure 2 shows the differences in organ function between the groups, with detailed results available in the supplementary material (Additional file: Table S1). Parameters related to liver, renal, and cardiac function as well as the lactate level were significantly higher in the sepsis group, while the PaO_2_/FiO_2_ ratio was notably lower (353.3 vs. 427.5 mmHg). Although PLT was not significantly different between the two groups, there was a trend toward higher levels in the non‐sepsis group (175 × 10^9^/L vs. 155 × 10^9^/L).

**Figure 3:**
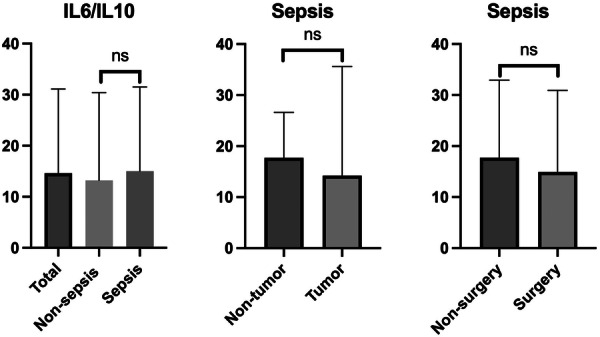
Distribution of IL6/IL10 ratio among different groups and subgroups. Abbreviation: IL, interleukin. The median values and 75th percentiles are shown. Outliers were removed. ns: *p* > 0.05.

We used the IL6/IL10 ratio to assess the balance between pro‐inflammatory and anti‐inflammatory responses in each patient. As shown in Figure 3, there was no significant difference in the IL6/IL10 ratio between the sepsis and non‐sepsis groups. Further analysis within the sepsis group revealed that the IL6/IL10 ratio was also not significantly different between patients with and without tumors or between patients who did and did not undergo surgery (Additional file: Table S2).

Both univariate and multivariate models were used to assess the correlations between the IL6/IL10 ratio and the clinical outcomes (Table 2). In the multivariate model, we adjusted for sex, age, and APACHE II scores. The results showed significant correlations only in the sepsis group, in both the univariate and multivariate models. Higher IL6/IL10 ratios were associated with shorter hospital stays, shorter ICU stays, and reduced MVt.

**Table 2:** Regression models between IL6/IL10 ratio and clinical outcomes of the study population.

		OR/β value and 95% CI		
Model	Outcomes	Total *N* = 208	Non‐sepsis *N* = 91(43.7%)	Sepsis *N* = 117(56.3%)
Single‐factor model	LOS (days)	**−0.515 (−0.643, −0.387)**	1.500 (**−**4.248, 7.247)	**−0.525 (−0.650, −0.399)**
	ICU stay (days)	**−0.181 (−0.246, −0.116)**	**−**0.442 (**−**0.993, 0.108)	**−0.180 (−0.245, −0.115)**
	MVt (hours)	**−1.118 (−2.189, −0.046)**	**−**0.831 (**−**11.132, 9.469)	**−1.120 (−2.194, −0.044)**
Muti‐factor model	LOS (days)	**−0.526 (−0.684, −0.369)**	0.680 (**−**5.975, 7.335)	**−0.535 (−0.704, −0.367)**
	ICU stay (days)	**−0.183 (−0.245, −0.121)**	**−**0.557 (**−**1.183, 0.068)	**−0.181 (−0.250, −0.112)**
	MVt (hours)	**−1.510 (−2.565, −0.456)**	**−**2.228 (**−**14.624, 10.167)	**−1.505 (−2.709, −0.301)**

*Note:* Bold values indicate statistically significant *p* < 0.05.

Abbreviations: CI, confidence interval; ICU, intensive care unit; IL, interleukin; LOS, length of stay; MVt, mechanical ventilation time; OR, odds ratio.

The secondary outcomes of the study were organ functions. As shown in Table 3, correlations between the IL6/IL10 ratio and organ functions were assessed using both univariate and multivariate models. In the univariate model, significant positive associations were found for all organ functions except PLT and PaO_2_/FiO_2_ in patients with sepsis. The β values and 95% confidence intervals for PLT and PaO_2_/FiO_2_ were −3.579 (− 4.336, −2.822) and −6.442 (− 8.458, −4.427), respectively. In the multivariate model, the correlation with liver function became insignificant, although the trend remained consistent with the univariate model.

**Table 3:** Regression models between IL6/IL10 ratio and organ function of the study population.

Model	Organ function	Total *N* = 208	Non‐sepsis *N* = 91 (43.7%)	Sepsis *N* = 117 (56.3%)
		*β* value and 95% CI		
Single‐factor model	PLT (×10^9^/L)	**−3.426 (−4.417, −2.435)**	27.180 (**−**33.622, 87.981)	**−3.579 (‐4.336, −2.822)**
	ALT (U/L)	1.152 (**−**0.024, 2.328)	**−**37.470 (**−**77.051, 2.111)	**1.345 (0.362, 2.329)**
	Cr (μmol/L)	**7.338 (6.442, 8.234)**	**−**17.300 (**−**39.583, 4.983)	**7.461 (6.483, 8.440)**
	BUN (mmol/L)	**0.356 (0.286, 0.425)**	**−**0.475 (**−**2.509, 1.560)	**0.360 (0.287, 0.433)**
	cTnI (μmol/L)	**120.858 (28.272, 213.444)**	**−**9507.64 (**−**28791.03, 9775.75)	**147.407 (82.025, 212.789)**
	NT‐proBNP (pg/ml)	**244.785 (178.211, 311.359)**	133.694 (**−**301.259, 568.647)	**245.027 (178.468, 311.587)**
	PaO_2_/FiO_2_ (mmHg)	**−6.438 (−8.507, −4.369)**	**−**5.618 (**−**109.286, 98.051)	**−6.442 (−8.458, −4.427)**
Muti‐factor model	PLT (×10^9^/L)	**−3.022 (−4.176, −1.869)**	23.549 (**−**39.448, 86.546)	**−3.389 (−4.341, −2.438)**
	ALT (U/L)	0.997 (**−0.500,** 2.494)	**−**41.760 (**−**84.820, 1.301)	1.228 (**−**0.210, 2.667)
	Cr (μmol/L)	**6.239 (5.141, 7.336)**	**−**22.001 (**−**44.340, 0.338)	**6.288 (5.020, 7.557)**
	BUN (mmol/L)	**0.295 (0.231, 0.359)**	**−**0.678 (**−**1.968, 0.613)	**0.300 (0.230, 0.370)**
	cTnI (μmol/L)	**176.169 (92.97, 259.37)**	2469.40 (**−**2541.30, 7480.10)	**187.44 (88.94, 285.94)**
	NT‐proBNP (pg/ml)	**213.391 (130.677, 296.106)**	239.135 (**−**141.317, 619.588)	**223.950 (144.485, 303.416)**
	PaO_2_/FiO_2_ (mmHg)	**−5.934 (−8.593, −3.276)**	**−**2.336 (**−**115.751, 111.079)	**−5.964 (−8.620, −3.308)**

*Note:* Bold values indicate statistically significant *p* < 0.05.

Abbreviations: ALT, alanine aminotransferase; BUN, blood urea nitrogen; CI, confidence interval; Cr, creatinine; cTnI, cardiac troponin I; Lac, lactate; NT‐proBNP, N‐terminal pro‐B‐type natriuretic peptide; PLT, platelet count.

Because of the significant statistical differences in the correlation between the IL6/IL10 ratio and various organ functions in the sepsis group, we conducted a sensitivity analysis focusing on patients with sepsis. Tumors and surgery are two main factors that affect the inflammatory status; therefore, further analyses focused on these two factors. Table 4A presents the sensitivity analysis in patients with and without tumors. There were 80 patients without tumors in the sepsis group. Significant correlations were found in nearly every organ function in the non‐tumor group, whereas significant correlations were only found for the lactate level and PaO_2_/FiO_2_ in patients with tumors. The correlations were stronger in patients with than without tumors.

**Table 4:** Sensitivity analyses between IL6/IL10 ratio and organ function using multi‐factor model in sepsis population.

	*β* value and 95% CI	
Organ function	Non‐tumor (*N* = 80)	Tumor (*N* = 37)
A. Tumor and non‐tumor		
PLT (×10^9^/L)	**−3.424 (−4.142, −2.706)**	61.572 (**−**46.632, 169.776)
ALT (U/L)	0.950 (**−**0.911, 2.810)	42.830 (**−**26.831, 112.492)
Cr (μmol/L)	**6.243 (4.918, 7.568)**	**−**5.101 (**−**110.907, 100.705)
BUN (mmol/L)	**0.308 (0.241, 0.374)**	2.420 (**−**7.000, 11.840)
cTnI (μmol/L)	**193.264 (70.282, 316.245)**	**−**10519.28 (**−**24149.36, 3110.80)
NT‐proBNP (pg/ml)	**208.391 (108.737, 308.045)**	846.284 (**−**931.743, 2624.310)
PaO_2_/FiO_2_ (mmHg)	**−4.245 (−7.106, −1.384)**	**−291.907 (−486.379, −97.434)**
Lac (mmol/L)	**0.113 (0.074, 0.152)**	**3.085 (1.237, 4.934)**

	β value and 95% CI	
Organ function	Non‐surgery (*N* = 26)	Surgery (*N* = 91)
B. Surgery and non‐surgery		
PLT (×10^9^/L)	31.782 (**−**97.241, 160.806)	**−3.605 (−4.581, −2.628)**
ALT (U/L)	**−**9.775 (**−**67.597, 48.047)	1.201 (**−**0.467, 2.869)
Cr (μmol/L)	**175.135 (78.548, 271,721)**	**5.762 (4.315, 7.210)**
BUN (mmol/L)	4.818 (**−**12.046, 21.681)	**0.307 (0.240, 0.375)**
cTnI (μmol/L)	**−**1719.37 (**−**4730.30,1291.55)	**180.854 (68.819, 292.889)**
NT‐proBNP (pg/ml)	901.17 (**−**6693.33, 8495.67)	**217.246 (120.186, 314.307)**
PaO_2_/FiO_2_ (mmHg)	2.997 (**−**291.217, 297.210)	**−6.500 (−9.591, −3.410)**
Lac (mmol/L)	0.530 (**−**5.182, 6.243)	**0.130 (0.102, 0.158)**

Abbreviations: ALT, alanine aminotransferase; BUN, blood urea nitrogen; Cr, creatinine; cTnI, cardiac troponin I; Lac, lactate; NT‐proBNP, N‐terminal pro‐B‐type natriuretic peptide; PLT, platelet count. Bold: *p* < 0.05.

Table 4B shows the analyses conducted in patients who underwent surgery and those who did not. The majority of patients had undergone surgery; only 26 patients in the sepsis group did not. Significant correlations were observed in nearly every organ function (except liver function) in the surgery group. Creatinine was the only significant biomarker that was positively correlated with the IL6/IL10 ratio in both groups, with a much higher correlation coefficient in the non‐surgery group.

## 4 Discussion

This study assessed whether the IL6/IL10 ratio reflects the pro‐/anti‐inflammatory balance and is associated with organ dysfunction in critically ill patients, particularly those with sepsis. We found that a higher IL6/IL10 ratio was associated with worse coagulation, renal, cardiac, and pulmonary function in patients with sepsis, but not in those without sepsis, suggesting sepsis‐specific links between inflammation and organ injury. In sensitivity analyses, these associations were stronger in patients without tumors and in those who had undergone surgery, supporting the potential clinical utility of the IL6/IL10 ratio for organ‐function assessment in sepsis.

The inflammatory response is a key feature of critical illnesses such as sepsis and persists throughout the disease course [17]. The host inflammatory response depends on the balance between pro‐inflammatory and anti‐inflammatory processes, and an imbalance in this response may be a common mechanism in critical illnesses. Existing studies have confirmed that pro‐ and anti‐inflammatory responses during the course of sepsis may occur successively or coexist simultaneously. Therefore, this complex change determines that merely focusing on pro‐ or anti‐inflammatory responses may be one‐sided [18]. IL6 is a classical pro‐inflammatory factor, and elevated plasma IL6 levels are associated with more severe sepsis and can contribute to organ dysfunction [19, 20]. The anti‐inflammatory response typically accompanies the pro‐inflammatory response, and IL10 is a crucial anti‐inflammatory cytokine that plays an essential role in maintaining organ function during inflammation [21]. The interaction between IL6 and IL10, represented by the IL6/IL10 ratio, serves as an important indicator of the balance between pro‐inflammatory and anti‐inflammatory responses [14]. Based on these concepts, this study explored the role and significance of IL6/IL10 in assessing organ function in critically ill patients. Our study analyzed the serum concentrations of IL6 and IL10 in both patients with and without sepsis and found no statistically significant difference in the IL6/IL10 ratio between the two groups. However, the IL6 levels were significantly higher than the IL10 levels in both groups. An increase in the IL6/IL10 ratio suggests a stronger pro‐inflammatory than anti‐inflammatory response. This result may be influenced by the proportion of postoperative patients in the non‐septic group. In patients who have experienced trauma or undergone major surgery, the inflammatory response is often significantly impacted, leading to elevated serum IL6 levels [22]. Additionally, not all patients with sepsis included in this study were in the early stage of the disease, which could have caused variability in pro‐inflammatory and anti‐inflammatory levels. This might explain the lack of significant differences in the IL6/IL10 ratio between the two groups.

Organ dysfunction is another key feature of critical illnesses [17], and sepsis, as a classic example of a critical illness, frequently leads to organ dysfunction. Our study showed that compared to patients without sepsis, those with sepsis often experienced dysfunction of the liver, kidneys, heart, and lungs. In critically ill patients, organ dysfunction is closely associated with the host inflammatory response, and an imbalance between pro‐inflammatory and anti‐inflammatory responses increases the risk of organ dysfunction. Understanding the relationship between the pro‐inflammatory/anti‐inflammatory balance and organ function may aid in improving the management of critical illnesses such as sepsis [13]. Most studies on the IL6/IL10 ratio have focused on tumors and polytrauma, with relatively few examining sepsis and even fewer exploring septic organ dysfunction [18, 23]. Therefore, in the present study, we investigated the relationship between IL6/IL10 and organ dysfunction in critical illnesses, particularly in sepsis. Although receiver operating characteristic curves are standard for evaluating predictive value, we did not perform them in this study. The IL6/IL10 ratio was treated primarily as a continuous variable to examine its association with organ function, rather than as a dichotomous predictive tool for binary outcomes. Studies have shown that IL6/IL10 is significantly correlated with organ function in ICU patients. However, this correlation was observed only in patients with sepsis; no significant correlation was found between IL6/IL10 and organ function in patients without sepsis. This finding highlights the differences in the pathological mechanisms between patients with and without sepsis. Sepsis is characterized by life‐threatening organ dysfunction due to a dysregulated host response to infection, often involving an abnormal inflammatory response, with an imbalance between pro‐inflammatory and anti‐inflammatory processes [5]. In other words, patients with sepsis develop a more intense inflammatory response than do patients without sepsis, and this imbalance in the host response plays a crucial role in the onset and progression of organ dysfunction [12].

An increase in the IL6/IL10 ratio is associated with elevated creatinine and BUN, increased cTnI and NT‐proBNP, and reduced PLT and PaO_2_/FiO_2_. These changes suggest that an elevated IL6/IL10 ratio may contribute to renal, cardiac, coagulation, and lung dysfunction. The host inflammatory response is linked to the severity of renal dysfunction in typical hemolytic uremic syndrome, where an increase in IL6/IL10 is closely related to a significant decline in the glomerular filtration rate [24]. Studies in patients undergoing hemodialysis have also shown a significantly increased IL6/IL10 ratio in those with regional left ventricular systolic dysfunction before dialysis [25]. Similarly, patients with severe chronic obstructive pulmonary disease can exhibit an impaired inflammatory state marked by a significantly increased IL6/IL10 ratio, indicating that IL6/IL10 is associated with lung dysfunction [26].

Liver injury can trigger the release of inflammatory factors. A study of patients undergoing hepatectomy showed that the IL6/IL10 ratio may better reflect the balance between pro‐inflammatory and anti‐inflammatory responses, suggesting a possible association with liver dysfunction [27]. However, our study did not show a correlation between IL6/IL10 and ALT. One possibility is that IL6/IL10 may not be a prominent marker for liver function. Another possibility is that we did not include other liver function parameters, such as bilirubin, which may have a stronger correlation with IL6/IL10.

Few studies have explored the relationship between IL6/IL10 and coagulation function. Our study suggests that an increase in IL6/IL10 is associated with deterioration in coagulation function in critically ill patients, which could provide a valuable addition to existing research. Some studies have also shown that increased IL10 levels are associated with organ dysfunction, but many of these studies did not clearly show changes in the IL6/IL10 ratio [28, 29]. Furthermore, in our observational cohort, the exact biological mechanisms underlying the differential correlations between the IL6/IL10 ratio and specific organ dysfunctions remain unclear, which warrants further investigation in future mechanistic studies.

Poor clinical outcomes are another important characteristic of critical illnesses such as sepsis [17]. In our study, sepsis patients had longer ICU stays and MVts than that for patients without sepsis, consistent with findings reported in the literature. Inflammation is closely related to organ dysfunction and may influence patients' prognosis. As previously reported, inflammatory markers such as IL6 and IL10 have been emphasized for their potential to predict clinical outcomes [16, 30]. Therefore, it is important to further explore the relationship between IL6/IL10 and prognosis in critically ill patients.

Our results suggest that only in patients with sepsis, IL6/IL10 was negatively correlated with the length of hospital stay, ICU stay, and MVt. These findings differ from previous studies in patients with polytrauma and coronavirus disease 2019 (COVID‐19). Sapan et al. [14] found that patients with polytrauma and an elevated IL6/IL10 ratio were more likely to experience mortality. Similarly, McElvaney et al. [31] showed that the IL6/IL10 ratio in patients with COVID‐19 was significantly correlated with worse clinical outcomes. One reason for these differences could be the distinct patient populations included in the studies. Our study focused on patients with sepsis in the ICU, whereas the others included patients with polytrauma or COVID‐19, which may lead to different effects of inflammation on prognosis.

Another possible explanation is that patients with an elevated IL6/IL10 ratio may be more prone to organ dysfunction and severe clinical manifestations, leading clinicians to observe and monitor these patients more closely. Inflammation may be only one of many factors that influence patient outcomes, and the effects of close monitoring and active treatment on prognosis cannot be overlooked. Unfortunately, the patient population in our study does not support further investigation into this mechanism. Future research should involve a larger, multicenter cohort study to explore this relationship more comprehensively.

Given that both surgery and the presence of a tumor can affect IL6/IL10 levels, we performed sensitivity analyses in patients with sepsis. We found that IL6/IL10 correlated more strongly with organ function in patients with sepsis who did not have tumors, whereas in patients with tumors, IL6/IL10 was only negatively correlated with PaO_2_/FiO_2_. This finding may be due to the unique inflammatory response seen in patients with tumors. Inflammatory responses are closely linked to all stages of tumor development and malignant progression [32]. Unlike patients with infection or sepsis alone, those with tumors often experience chronic inflammation. Chronic inflammation contributes to immunosuppression, creating a microenvironment that supports tumor initiation, growth, and metastasis [33].

Chronic, disordered, and persistent inflammation is also associated with an increased risk of malignancy and the progression of most tumor types [34]. Additionally, unlike trauma or infection, the inflammatory response in patients with tumors cannot be fully eliminated [35]. However, exogenous inflammation caused by bacterial or viral infections can increase tumor risk and accelerate malignant progression [36]. Despite this, chronic inflammation in patients with tumors often does not lead to acute organ dysfunction. Therefore, patients with both sepsis and tumors exhibit a highly complex inflammatory response, which may influence the relationship between IL6/IL10 and organ function indicators.

This study also showed that IL6/IL10 was more strongly correlated with organ function in patients with sepsis who had undergone surgery, while IL6/IL10 was only positively correlated with creatinine in those without surgery. This result is understandable because trauma from surgery can activate an early systemic pro‐inflammatory response, leading to the increased production of pro‐inflammatory cytokines [37]. This pro‐inflammatory response may in turn trigger a systemic anti‐inflammatory response, resulting in immunosuppression caused by surgical trauma [38]. Studies have shown that patients admitted to the ICU shortly after surgery often exhibit both a systemic pro‐inflammatory response and immunosuppression [39]. For instance, trauma from procedures such as total knee replacement triggers systemic inflammatory responses, elevating the IL6 and IL10 levels; the IL6/IL10 ratio is correlated with trauma severity and indicates pro‐inflammatory or anti‐inflammatory dominance [40, 41]. Similarly, in patients undergoing thoracic surgery, the plasma IL6 and IL10 concentrations are significantly higher at wound closure and on the first postoperative day [42]. As with sepsis, the early postoperative inflammatory response may also contribute to organ dysfunction [43].

In patients with pulmonary dysfunction following major abdominal surgery, the IL6 levels were significantly elevated on the first postoperative day [43]. Similarly, elevated IL6 and IL10 levels were observed in patients with pulmonary dysfunction after esophagectomy [44]. Therefore, patients with sepsis who have undergone surgery tend to develop a stronger early inflammatory response, which may lead to organ dysfunction. This connection between inflammation and organ dysfunction is reflected in the correlation between IL6/IL10 and various organ function parameters.

Some limitations of this study should be noted. First, the small sample size limited our ability to conduct an in‐depth analysis of the relationship between the serum IL6 and IL10 concentrations and the prognosis of critical illnesses such as sepsis. Therefore, the clinical value of the IL6/IL10 ratio in predicting the prognosis needs to be confirmed in larger cohort studies. For future research, we plan to continue collecting relevant cases to further validate our findings. Second, this study was conducted at a single center, and the relatively low mortality in our patient population prevented a more detailed analysis of mortality. High‐quality, multicenter cohort studies are needed to further explore the role and value of IL6/IL10 in predicting prognosis in patients with sepsis. Thirdly, the patients in the non‐sepsis group exhibit potential heterogeneity, which may weaken the statistical comparison results of the inflammatory responses between the sepsis group and the non‐sepsis group. Finally, although we conducted a sensitivity analysis in patients with sepsis, the number of patients with tumors and those without surgery was relatively small. Consequently, interpretations drawn from these subgroups should be considered exploratory. Future studies should include more of these patients to confirm the conclusions of this study.

## 5 Conclusion

Our study showed that in critical illnesses, particularly sepsis, the IL6/IL10 ratio is significantly associated with outcomes such as organ dysfunction, including dysfunction in coagulation, kidneys, heart, and lungs. Sensitivity analysis in patients with sepsis indicated that IL6/IL10 was more strongly associated with organ dysfunction in postoperative patients and those without tumors. These findings are primarily hypothesis‐generating and contribute to the understanding of the role of IL6/IL10 in septic organ dysfunction, suggesting that the IL6/IL10 ratio may serve as a valuable marker associated with inflammation and the severity of critical illness, which warrants further prognostic validation in larger cohorts.

## Author Contributions

**Guang‐Jian Wang:** investigation, resources, writing – original draft. **Hui Lian:** formal analysis, writing – original draft, writing – review and editing. **Hong‐Min Zhang:** writing – review and editing. **Ye‐Cheng Liu:** conceptualization, writing – review and editing. **Xiao‐Ting Wang:** conceptualization, writing – review and editing.

## Ethics Statement

This study was approved by the ethics committee of Peking Union Medical College Hospital, Beijing, China (Approval No. I‐22PJ1072). Written informed consent was obtained from the patients or their relatives Written informed consent was obtained from all participating patients prior to enrollment. For patients who were unconscious or otherwise lacked the capacity to provide direct consent due to the severity of their illness, written informed consent was obtained from their legally authorized representatives or next of kin.

## Consent

The authors have nothing to report.

## Conflicts of Interest

The authors declare no conflicts of interest.

## Supporting information

Additional file.

## Data Availability

All datasets used and analyzed during the current study are available from the corresponding author on reasonable request.
