# Supplementary material for: Relationship Between IL6/IL10 Serum Concentrations and Organ Function in Critically Ill Patients Based on Sepsis: A Prospective Study
Source: Immun Inflamm Dis. 2026 Apr 9;14(4):e70414. doi: 10.1002/iid3.70414 (PMC13066732; doi:10.1002/iid3.70414)
Supplement: Supplementary file 1 — Additional file. [file IID3-14-e70414-s001.docx]

Table S1: Organ function of the study population.

|  | Total  N=208 | | | Non-sepsis  N=91 | | | Sepsis  N=117 | | | P-value |
| --- | --- | --- | --- | --- | --- | --- | --- | --- | --- | --- |
|  | Medium | 25^th^ | 75^th^ | Medium | 25^th^ | 75^th^ | Medium | 25^th^ | 75^th^ |  |
| PLT (×10^9^/L) | 166 | 119 | 217 | 175 | 131 | 226 | 155 | 115 | 209 | 0.061 |
| ALT (U/L) | 16 | 10 | 32 | 14.0 | 8.5 | 24.5 | 18 | 12 | 34 | 0.013 |
| Cr (μmol/L) | 62 | 47 | 97 | 56.0 | 46.0 | 73.5 | 70.5 | 48 | 111.5 | <0.001 |
| BUN (mmol/L) | 4.92 | 3.30 | 7.31 | 3.7 | 2.8 | 5.6 | 6.2 | 4.2 | 9.6 | <0.001 |
| cTnI (μmol/L) | 32 | 9 | 1588 | 12 | 5 | 200 | 163.5 | 16 | 3550.2 | 0.008 |
| NT-proBNP (pg/ml) | 353 | 163 | 1701 | 238 | 126 | 353 | 911.0 | 200.8 | 2834.5 | <0.001 |
| PaO_2_/FiO_2_ (mmHg) | 385.5 | 280.3 | 500 | 427.5 | 301.2 | 510.9 | 353.3 | 260.5 | 473.3 | 0.023 |
| Lac (mmol/L) | 1.7 | 1.0 | 3.5 | 1.5 | 0.9 | 2.2 | 2.0 | 1.1 | 4.0 | 0.003 |

Abbreviations: PLT, platelet count; ALT, alanine aminotransferase; Cr, creatinine; BUN, blood urea nitrogen; cTnI, cardiac troponin I; NT-proBNP, N-terminal pro-B-type natriuretic peptide; Lac, lactate. Bold: P < 0.05

Table S2: Distribution of IL6/IL10 among different groups and sub-groups.

1. Distribution of IL6/IL10 in sepsis and non-sepsis groups.

|  | Total  N=208 | | | Non-sepsis  N=91 | | | Sepsis  N=117 | | | P-value |
| --- | --- | --- | --- | --- | --- | --- | --- | --- | --- | --- |
|  | Medium | 25^th^ | 75^th^ | Medium | 25^th^ | 75^th^ | Medium | 25^th^ | 75^th^ |  |
| IL6/IL10 | 14.6 | 8.0 | 31.1 | 13.2 | 8.0 | 30.4 | 15.0 | 8.0 | 31.5 | 0.430 |

1. Distribution of IL6/IL10 in sub-groups of sepsis patients.

| N=117 | No | | | | Yes | | | | P-value |
| --- | --- | --- | --- | --- | --- | --- | --- | --- | --- |
|  | N | Medium | 25^th^ | 75^th^ | N | Medium | 25^th^ | 75^th^ |  |
| Septic Shock | 31 | 16.1 | 8.3 | 29.9 | 86 | 15.0 | 8.0 | 32.3 | 0.520 |
| Tumor | 80 | 17.7 | 8.1 | 26.6 | 37 | 14.2 | 7.5 | 35.6 | 0.551 |
| Surgery | 26 | 17.7 | 4.6 | 32.9 | 91 | 14.9 | 8.5 | 30.9 | 0.647 |

Abbreviation: IL, interleukin. The median values and 75th percentiles are shown. Outliers were removed.
